# Incidence and time trends of herpes zoster among patients with head and neck cancer who did and did not undergo radiotherapy: A population-based cohort study

**DOI:** 10.1371/journal.pone.0250724

**Published:** 2021-05-20

**Authors:** Peng-Yi Lee, Jung-Nien Lai, Lu-Ting Chiu, Yu-Ting Wei

**Affiliations:** 1 Department of Radiation Oncology, China Medical University Hospital, China Medical University, Taichung, Taiwan; 2 Department of Radiation Oncology, Lin Shin Hospital, Taichung, Taiwan; 3 School of Chinese Medicine, College of Chinese Medicine, China Medical University, Taichung Taiwan; 4 Department of Chinese Medicine, China Medical University Hospital, Taichung, Taiwan; 5 Management office for Health Data, China Medical University Hospital, Taichung, Taiwan; 6 College of Medicine, China Medical University, Taichung, Taiwan; 7 Preventive Medicine Center, Taichung Tzu Chi Hospital, Buddhist Tzu Chi Medical Foundation, Taichung, Taiwan; 8 Division of Family Medicine, Department of Community Medicine, China Medical University Hospital, Taichung, Taiwan; Chang Gung Memorial Hospital and Chang Gung University, Taoyuan, Taiwan, TAIWAN

## Abstract

**Purpose:**

This study aimed to determine the risk and time trends of herpes zoster among patients with head and neck cancer, with or without radiotherapy.

**Methods:**

A total of 2160 patients with head and neck cancer were enrolled. The radiotherapy and non- radiotherapy cohorts were frequency-matched at a 1:1 ratio according to sex, age, and index date. Moreover, 1080 matched non-cancer individuals were considered normal controls. Data were obtained from the National Health Insurance Research Database and Cancer Registry. The primary end point was the incidence of herpes zoster, and the adjusted confounding factors were age, sex, comorbidities, oncological surgery, and chemotherapy.

**Results:**

The incidence of herpes zoster was higher in cancer patients than in non-cancer individuals but did not significantly differ (13.67 vs. 8.06 per 1,000 person-years, *p =* 0.18). The risk of herpes zoster was significantly higher in the radiotherapy cohort than in the non-radiotherapy cohort (18.55 vs. 9.06 per 1,000 person-years, *p =* 0.03). The 5-year incidence rates in the radiotherapy and non-radiotherapy cohorts were 8.9% and 5%, respectively (*p* < 0.0001). Survival analysis indicated there was no immortal time bias. The time trends in the radiotherapy cohort persistently showed a high risk within the first 2 years, which decreased thereafter. Only patients with comorbid rheumatoid arthritis showed a significantly high risk of herpes zoster (*p* = 0.02). Oncological surgery and chemotherapy had no impact on the development of herpes zoster.

**Conclusions:**

This nationwide population-based study showed that patients with head and neck cancer receiving radiotherapy are at an increased risk of herpes zoster. Health care professionals should pay more attention to this vulnerable group to improve their quality of life.

## Introduction

Herpes zoster (HZ) is a cutaneous disease caused by the reactivation of endogenous and latent varicella-zoster virus that is dormant in the cranial nerve or dorsal root ganglia [1]. It is characterized by dermatomal unilateral painful sores, which mainly appear on the chest and then on the head and neck (HN), waist, and thigh. However, it is less common on the four limbs, hands, or feet. This condition may cause long-term sequelae, including post-herpetic neuralgia, thereby deteriorating a patient’s quality of life (QoL) [2]. Moreover, only few individuals experience devastating complications like retinal necrosis, neuropathy, vasculopathy, or encephalitis. In the literature, for general population, the incidence of HZ is approximately 10%–20%, while up to 50% could be observed in certain high-risk individuals [3]. People with conditions of impaired immunity, including human immunodeficiency virus infection, organ transplantation, old age, and cancer-related treatments, such as chemotherapy (CT) and radiotherapy (RT)were found more susceptible to HZ infection [4]. Cancer itself is a preceding factor of HZ. Those with hematological malignancies had a higher possibilities of HZ development compared to ones with solid cancers [5–8]. For patients with a specific cancer diagnosis, the effect of different treatment modalities on risk of HZ has not been well understood [8–10].

The QoL of cancer patients has become a concern because rapid advancements in medical care have prolonged survival. The treatments for patients with HN cancers were usually a combination of surgery, RT, and CT. However, the populations and treatment modalities in previous studies were heterogeneous, and a well-controlled comparison was not performed. Therefore, quantifying the impact of RT alone on the risk of HZ is challenging, and data regarding HN cancers are limited [10, 11]. A nationwide population-based cohort study was designed to determine the risk of HZ development among HN cancer victims who did and did not undergo RT with adjustment for potential confounders. The current study primarily aimed to identify certain vulnerable groups and improve the QoL of cancer survivors.

## Materials and methods

### Data source and participants

We obtained patient data from the National Health Insurance Research Database (NHIRD), which was established in 1995. This is a single-payer system containing medical claims data and is compulsory for all Taiwanese citizens. The Longitudinal Health Insurance Database 2000, which is a subset of NHIRD, consisted of 1 million beneficiaries randomly selected in 2000. A registry of drug prescriptions, disease profiles, beneficiaries, and other medical services were included in this subset. The disease history of each insured individual was recorded according to the International Classification of Diseases, 9th Revision, Clinical Modification (ICD-9-CM). This study has been approved by the Research Ethics Committee at the China Medical University Hospital (CMUH104-REC2-115- CR-4).

The inclusion criteria were as follows: patients older than 30 years and with at least one diagnosis of HN cancer (ICD-9-CM code: 140, 141, 142, 143, 144, 145, 146, 147, 148, 149, 160, 161, 195.0, and 196.0, details shown in S1 Table) from 2000 to 2013 but without any other cancer diagnoses (ICD-9-CM code: 140–208) and without a history of HZ before the index date, which was defined as the initial date of RT. The enrolled patients were assigned into two groups (RT and non-RT cohorts) according to whether they had received RT or not after HN cancer was diagnosed. The non-RT cohort was frequency-matched with the RT cohort at a 1:1 ratio according to sex, age (every 5 years), and index date (S1 Fig). Another reference cohort was comprised of the same number of general populations without cancer and matched to be the normal control.

### Ethics statement

The NHIRD encrypts personal information of all subjects to protect their privacy and provides anonymous identification numbers to researchers. Therefore, patient consent is not required to access data from the NHIRD. The Institutional Review Board (IRB) of China Medical University (CMUH104-REC2-115-CR-4) approved this study to fulfill the condition for exemption, and informed consents from participants were waived.

### Outcome and potential confounders

The primary end point of this study was HZ (ICD-9-CM code 053.0–053.9) development in patients with HN cancer. The follow-up period was defined as the time from the index date until one of the following: the end of 2013, withdrawal from the NHI program, or the onset of HZ. Human immunodeficiency virus infection (HIV, ICD-9-CM: 042), diabetes (DM, ICD-9-CM code 250), hepatitis C (HCV, ICD-9 code 070.41, 070.44, 070.51, 070.54, 070.70, and 070.71), hepatitis B (HBV, ICD-9-CM code 070.20–070.33), rheumatoid arthritis (RA, ICD-9-CM code 714), systemic lupus erythematosus (SLE, ICD-9-CM code 710.0), hypertension (HTN, ICD-9-CM code 401–405), and chronic obstructive pulmonary disease and allied conditions (COPD, ICD-9-CM code 490–496) were considered as potential confounders. Additionally, the role of oncological surgery and CT were evaluated. Oncological surgery was defined as a radical operation, such as sinusectomy, maxillectomy, mandibulectomy, glossectomy, or laryngopharyngectomy with and without lymph node dissection/lymphadenectomy. It should be noted that simple excisions for early-stage tumors and endoscopic surgery were excluded. CT was documented for patients who received any one of the commonly used drugs for head and neck cancer, including cytotoxic agents and target therapy. The time trends of HZ development were evaluated according to different follow-up periods.

Furthermore, to identify the association between RT and HZ with complications, the ICD-9-CM codes of HZ were divided into two groups. One was HZ with complications in the central nervous system or HN region (053.0, 053.11, 053.12, 053.20, 053.21, 053.22, 053.29, and 053.71), whereas the other one was HZ with other complications or without complications (053.10, 053.13, 053.19, 053.79, 053.8, and 053.9) (S2 Table).

### Statistical analysis

The SAS statistical software version 9.4 (SAS Institute Inc., Cary, NC, the USA) was used. Categorical and continuous variables were examined using the chi-square test and the independent *t*-test, respectively. The incidence of HZ in the two cohorts adjusted for potential confounders was compared using the Cox proportional hazards regression model. The survival time between the two cohorts were analyzed to determine an immortal time bias. The differences were assessed using the Kaplan–Meier method and the log-rank test. A two-tailed *p*-value of <0.05 was considered significant.

## Results

In total, 2982 patients met the inclusion criteria. Of them, the RT cohort was composed of 1080 patients receiving RT, and another 1080 patients were matched to be the non-RT cohort. The baseline characteristics are presented in Table 1. The median age of the participants was 56 years (*p* = 0.98). The demographic data was well balanced except that a higher proportion of patients had HTN in the non-RT cohort than in the RT cohort (40% vs. 45%, *p* = 0.02). No significant difference in median follow-up times was observed between the two cohorts (2.17 vs 2.36 years, *p* = 0.19). For treatment-related factors, the proportion of patients undergoing oncological surgery or CT was significantly higher in the RT cohort than in the non-RT cohort (54.91% vs. 29.35%, *p* < 0.0001; 72.04% vs. 9.07%, *p* < 0.0001).

**Table 1:** Demographic data and treatment-related factors in the RT and non-RT cohorts.

	Non-radiotherapy^a^ (n = 1080)		Radiotherapy (n = 1080)		
Characteristics	n	%	n	%	p-value
**Age, years**					0.98
<50	329	30.46	333	30.83	
50–65	481	44.54	478	44.26	
>65	270	25.00	269	24.91	
Median (IQR)	56.00 (48.27, 64.99)		55.95 (48.23, 64.93)		
**Sex**					1.00
Female	105	9.72	105	9.72	
Male	975	90.28	975	90.28	
**Comorbidity**					
Hypertension	486	45.00	432	40.00	0.02
Diabetes mellitus	273	25.28	241	22.31	0.11
Hepatitis B	44	4.07	45	4.17	0.91
Hepatitis C	27	2.50	24	2.22	0.67
HIV	2	0.19	1	0.09	0.56
Systemic lupus erythematosus	2	0.19	1	0.09	0.56
Rheumatoid arthritis	33	3.06	26	2.41	0.36
COPD	326	30.19	286	26.48	0.06
**Oncological surgery**					< .0001
No	763	70.65	487	45.09	
Yes	317	29.35	593	54.91	
**Chemotherapy drugs**					< .0001
No	982	90.93	302	27.96	
Yes	98	9.07	778	72.04	
**Follow-up, years** Median (IQR)	2.17 (0.91, 4.27)		2.36 (0.56, 4.37)		0.19

COPD, chronic obstructive pulmonary disease; HIV, human immunodeficiency virus; IQR, interquartile range; RT, radiotherapy; Data shown as n (%) or median (IQR);

^a^Using 1:1 frequency matching

**Fig 1:**
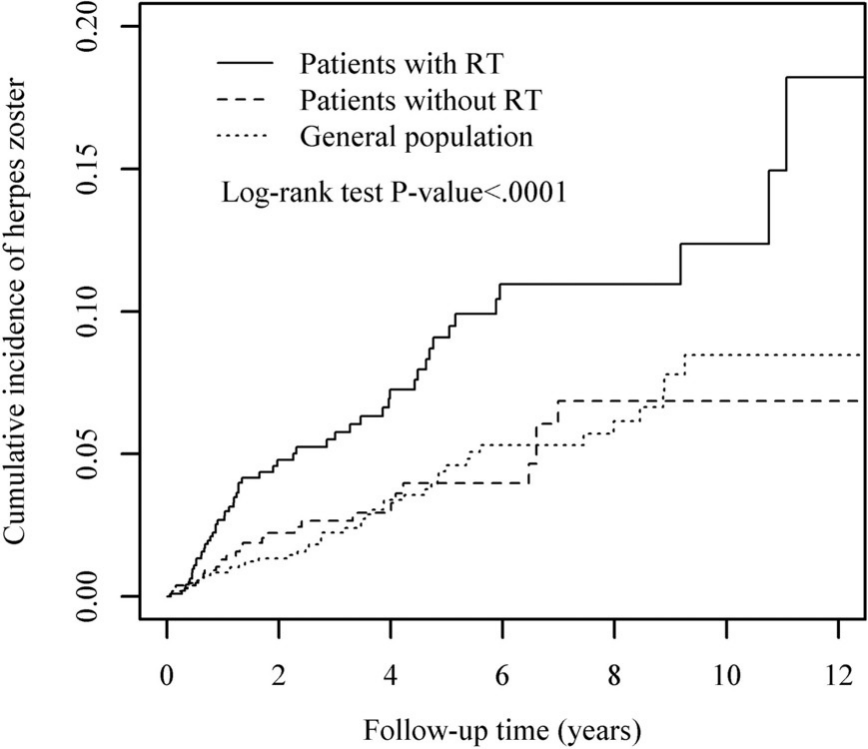
Cumulative incidence rates of herpes zoster in the radiotherapy cohort, non-radiotherapy cohort, and general population. RT, radiotherapy.

As shown in S3 Table and Fig 1, the incidence rates of HZ in non-cancer individuals (n = 1080) and in HN cancer patients as a whole (n = 2160) were 8.06 and 13.67 per 1000 person-years (PY) (adjusted hazard ratio [aHR]: 1.27; 95% confidence interval [CI]: 0.82–2.00, *p =* 0.18), respectively. Overall, the risk of HZ in patients with HN cancer was not significantly higher than that in the general population. Moreover, there was no statistically significant difference between the two cancer cohorts and the normal control. However, RT had a significant impact on HZ development among patients with HN cancer. The cumulative incidence rates of HZ in the RT and non-RT cohorts were 18.55 and 9.06 per 1,000 PY, respectively (aHR: 1.79, *p =* 0.03). The 2- and 5-year actuarial incidence rates in those receiving RT or not were 4.8% versus 1.7% and 8.9% versus 5%, respectively (*p <* 0.0001).

The HRs of HZ associated with baseline characteristics and treatment-related factors among patients with HN cancer are summarized in Table 2. There was 1.79-fold higher HZ risk in the RT cohort than in the non-RT cohort (95% CI: 1.02–2.97, *p =* 0.03) after adjustment for relevant confounding factors. In addition, individuals with RA had a higher risk of HZ than those without RA (38.71 vs. 13.03 per 1,000 PY, respectively; aHR: 2.87, 95% CI: 1.22–6.77, *p =* 0.02). Notably, oncological surgery or CT was not associated with HZ development.

**Table 2:** Univariate and multivariate analyses of confounding factors associated with herpes zoster.

Variables	Herpes zoster (n = 85)			Crude HR (95% CI)	*p*-value	Adjusted HR (95% CI)	*p*-value
	Event	PY	IR				
**Radiotherapy**							
No	29	3201	9.06	1 (reference)		1 (reference)	
Yes	56	3019	18.55	2.10 (1.35–3.30)*	0.001	1.79 (1.02–2.97)*	0.03
**Age, years**							
<50	22	1970	11.17	1 (reference)		1 (reference)	
50–65	33	2762	11.95	1.08 (0.63–1.85)	0.77	0.96 (0.54–1.69)	0.89
>65	30	1488	20.16	1.82 (1.05–3.16)*	0.03	1.69 (0.93–3.01)	0.10
**Sex**							
Female	8	644	12.42	1 (reference)		1 (reference)	
Male	77	5576	13.81	1.03 (0.51–2.20)	0.86	1.20 (0.57–2.52)	0.62
**Comorbidity**							
Hypertension							
No	44	3779	11.64	1 (reference)		1 (reference)	
Yes	41	2441	16.80	1.40 (0.91–2.15)	0.12	1.18 (0.73–1.93)	0.48
Diabetes mellitus							
No	65	4894	13.28	1 (reference)		1 (reference)	
Yes	20	1326	15.08	1.10 (0.66–1.82)	0.70	0.95 (0.56–1.61)	0.85
Hepatitis B							
No	82	6027	13.61	1 (reference)		1 (reference)	
Yes	3	193	15.54	1.09 (0.34–3.44)	0.89	1.16 (0.36–3.75)	0.80
Hepatitis C							
No	84	6092	13.79	1 (reference)		1 (reference)	
Yes	1	129	7.75	0.55 (0.08–3.96)	0.55	0.47 (0.06–3.53)	0.47
Systemic lupus erythematosus							
No	85	6205	13.70	1 (reference)		1 (reference)	
Yes	0	15	0.00	—		—	
Rheumatoid arthritis							
No	79	6065	13.03	1 (reference)		1 (reference)	
Yes	6	155	38.71	2.94 (1.28–6.75)*	0.01	2.87 (1.22–6.77)*	0.02
COPD							
No	55	4582	12.00	1 (reference)		1 (reference)	
Yes	30	1639	18.30	1.52 (0.98–2.39)	0.06	1.28 (0.79–2.10)	0.31
**Oncological surgery**							
No	37	3278	11.29	1 (reference)		1 (reference)	
Yes	48	2942	16.32	1.49 (0.97–2.29)	0.07	1.23 (0.78–1.93)	0.35
**Chemotherapy drugs**							
No	42	4012	10.47	1 (reference)		1 (reference)	
Yes	43	2208	19.47	1.85 (1.20–2.83)*	0.005	1.47 (0.86–2.50)	0.15

CI, confidence interval; COPD, chronic obstructive pulmonary disease; HN, head and neck; HR, hazard ratio; IR, incidence rate, per 1000 PY; PY, person-years; aHR adjusted for age, sex, hypertension, diabetes mellitus, hepatitis B, hepatitis C, systemic lupus erythematosus, rheumatoid arthritis, COPD, oncological surgery and chemotherapy drugs;

*p < 0.05

The HRs of RT-associated HZ, which were stratified based on age, sex, comorbidities, oncological surgery, and CT, were depicted in Table 3. RT was related to a significantly increased risk of HZ in the subgroup of patients with HTN (aHR = 2.2; 95% CI: 1.02–4.77, *p =* 0.02), those without HBV (aHR = 1.74; 95% CI: 1.02–2.94, *p =* 0.04), those without HCV (aHR = 1.76; 95% CI: 1.06–3.12, *p =* 0.02), those without SLE (aHR = 1.78; 95% CI: 1.02–2.96, *p =* 0.04), those without RA (aHR = 1.82; 95% CI: 1.07–3.43, *p =* 0.01), those without COPD (aHR = 2.63; 95% CI: 1.23–5.66, *p =* 0.008), and male patients (aHR = 1.86; 95% CI: 1.09–3.07, *p =* 0.02).

**Table 3:** Univariate and multivariate analyses of the effects of RT stratified by age, sex, comorbidities, oncological surgery, and chemotherapy drugs.

Variables	Radiotherapy						Crude HR (95% CI)	*p*-value	Adjusted HR (95% CI)	*p*-value
	No			Yes						
	Event	PY	IR	Event	PY	IR				
**All**	29	3201	9.06	56	3019	18.55	2.10 (1.35–3.30)*	0.001	1.79 (1.02–2.97)*	0.03
**Age, years**										
<50	7	989	7.08	15	980	15.31	2.27 (0.93–5.57)	0.07	1.77 (0.12–5.82)	0.27
50–65	8	1385	5.78	25	1376	18.17	3.23 (1.45–7.17)*	0.04	2.53 (0.89–5.97)	0.09
>65	14	826	16.95	16	662	24.17	1.39 (0.68–2.86)	0.36	1.52 (0.66–3.44)	0.33
**Sex**										
Female	4	343	11.66	4	301	13.29	1.07 (0.26–4.31)	0.91	0.93 (0.16–5.33)	0.94
Male	25	2858	8.75	52	2718	19.13	2.25 (1.40–3.63)*	0.0008	1.86 (1.09–3.07)*	0.02
**Comorbidity**										
Hypertension										
No	14	1896	7.38	30	1882	15.94	2.19 (1.17–4.14)*	0.02	1.25 (0.82–4.98)	0.21
Yes	15	1304	11.50	26	1137	22.87	2.10 (1.11–3.98)*	0.02	2.20 (1.02–4.77)*	0.04
Diabetes mellitus										
No	22	2453	8.97	43	2441	17.62	2.03 (1.22–3.40)*	0.007	1.70 (0.94–3.13)	0.10
Yes	7	747	9.37	13	578	22.49	2.72 (1.04–7.19)*	0.04	1.91 (0.88–6.75)	0.11
Hepatitis B										
No	29	3093	9.38	53	2933	18.07	1.98 (1.26–3.11)*	0.003	1.74 (1.02–2.94)*	0.04
Yes	0	107	0.00	3	86	34.88	—		—	
Hepatitis C										
No	28	3133	8.94	56	2958	18.93	2.18 (1.38–3.43)*	0.0008	1.76 (1.06–3.12)*	0.01
Yes	1	67	14.93	0	61	0.00	—		—	
Systemic lupus erythematosus										
No	29	3187	9.10	56	3018	18.56	2.10 (1.34–3.29)*	0.001	1.78 (1.02–2.96)*	0.04
Yes	0	14	0.00	0	1	0.00	—		—	
Rheumatoid arthritis										
No	26	3100	8.39	53	2965	17.88	2.21 (1.38–3.53)*	0.001	1.82 (1.07–3.43)*	0.01
Yes	3	101	29.70	3	54	55.56	1.79 (0.35–9.12)	0.48	1.45 (0.15–12.42)	0.65
COPD										
No	12	2301	5.22	43	2280	18.86	3.71 (1.95–7.04)*	< .0001	2.63 (1.23–5.66)*	0.008
Yes	17	899	18.91	13	739	17.59	0.93 (0.45–1.93)	0.86	1.00 (0.42–2.39)	0.99
**Oncological surgery**										
No	18	2130	8.45	19	1147	16.56	2.02 (1.06–3.85)*	0.03	1.28 (0.78–2.90)	0.28
Yes	11	1070	10.28	37	1872	19.76	1.93 (0.99–3.80)	0.05	1.92 (0.88–4.16)	0.09
**Chemotherapy drugs**										
No	27	2959	9.12	15	1052	14.26	1.61 (0.85–3.04)	0.14	1.48 (0.65–3.21)	0.35
Yes	2	241	8.30	41	1967	20.84	2.63 (0.64–10.87)	0.18	2.49 (0.59–10.47)	0.21

CI, confidence interval; COPD, chronic obstructive pulmonary disease; HR, hazard ratio; IR, incidence rate, per 1000 PY; PY, person-years; RT, radiotherapy; aHR adjusted for age, sex, hypertension, diabetes mellitus, hepatitis B, hepatitis C, systemic lupus erythematosus, rheumatoid arthritis, COPD, oncological surgery and chemotherapy drugs.

*p < 0.05

Table 4 presents the incidences of HZ in both cohorts according to different follow-up periods. The incidence of HZ remained steadily high within the first 2 years in the RT cohort (20.66 per 1000 PY within 6 months, 24.84 per 1000 PY between 6 and 12 months, and 22.94 per 1000 PY between 1 and 2 years) and then decreased thereafter (13.37 per 1000 PY after 2 years). During the follow-up period, the incidence of HZ was persistently higher in the RT cohort than in the non-RT cohort; however, the difference was not statistically significant in the four time periods, respectively. The highest incidence in the RT cohort was observed within 6–12 months after RT. Nevertheless, the increase associated with RT was evident within 6 months and 1–2 years after RT.

**Table 4:** Incidence of herpes zoster in non-RT and RT cohorts according to different follow-up periods.

Follow-up time	Radiotherapy						IR difference	Crude HR (95% CI)	*p*-value	Adjusted HR (95% CI)	*p*-value
	No			Yes							
	Event	PY	IR	Event	PY	IR					
**All**	29	3201	9.06	56	3019	18.55	9.49	2.10 (1.35–3.30)*	0.001	1.79 (1.02–2.97)*	0.03
<6 months	4	506	7.91	10	484	20.66	12.75	2.65 (1.03–8.45)*	0.03	2.55 (0.93–8.16)	0.06
6–12 months	8	501	15.97	12	483	24.84	8.87	1.55 (0.64–3.79)	0.33	1.80 (0.72–4.51)	0.21
1–2 years	6	616	9.74	12	523	22.94	13.2	2.34 (0.98–6.24)	0.05	2.46 (0.92–6.84)	0.08
>2 years	11	1646	6.68	22	1646	13.37	6.69	2.02 (0.98–4.17)	0.06	1.82 (0.89–3.73)	0.09

CI, confidence interval; COPD, chronic obstructive pulmonary disease; HR, hazard ratio; IR, incidence rate, per 1000 PY; PY, person-years.

*p < 0.05; aHR adjusted for age, sex, hypertension, diabetes mellitus, hepatitis B, hepatitis C, systemic lupus erythematosus, rheumatoid arthritis, COPD, oncological surgery and chemotherapy drugs

Survival analysis was carried out to determine an immortal time bias, which could impact the interpretation of our results. Patients in the RT cohort had a significantly lower survival than those in the non-RT cohort (5-year overall survival: 67% vs 94%, *p* < 0.0001) (S2 Fig).

As to the association between RT and HZ with complications, the events in the group of HZ with complications in CNS and HN region were extremely rare. There were only three events in the RT cohort and no event in the non-RT cohort. The details of this analysis are presented in S4 Table.

## Discussion

To the best of our knowledge, this is the first nationwide population-based study that assessed the risk of HZ in patients with HN cancer. In general, there was no significant difference in the risk of HZ between cancer patients and age- and sex-adjusted individuals who did not have cancer diagnoses. Among those with HN cancer, RT significantly impacted the development of HZ, with 2-year and 5-year actuarial incidence rates of 4.8% and 8.9%, respectively. Of note, the incidence of HZ in the RT cohort remained steady within the first 2 years, and then it decreased thereafter. Considering the complexity of treatment for HN cancer, the effect of oncological surgery, RT, and CT were analyzed in this study. Our findings indicated the importance of early monitoring for the occurrence of HZ in patients who receive RT. Moreover, the incidence of HZ was significantly high in individuals with RA and was relatively high in those who were older than 65 years old. Hence, the potential toxicities should be kept in mind by physicians for these vulnerable groups to improve their QoL.

In order to clarify that if there was immortal time bias in our cohorts, we performed survival analysis using the Kaplan–Meier method. The RT cohort had a significantly lower survival (5-year overall survival: 67% vs 94%, *p* < 0.0001). However, the incidence of HZ was still higher in the RT cohort than in the non-RT cohort. This result added credibility to our conclusion, since immortal time bias did not exist. Another evidence to support the absence of an immortal time bias in our cohort is the median follow-up time, which was not significantly different between the two groups.

Several studies have assessed the role of RT; however, the results have been controversial. A recent research showed that cancer patients receiving RT had a significantly higher risk of HZ (aHR: 2.59) compared with those who did not, particularly within the radiation field. Of note, HN cancers accounted for a small proportion in their cohort, and there was no difference in terms of the risk of HZ between them (18.5 vs. 5.8 per 1000 PYs for RT vs. non-RT group in HN cancers, *p* = 0.126) [9]. Another study focusing on patients with Hodgkin’s disease showed that the radiation field mattered. Those receiving limited-field RT had half the risk of HZ compared to those with extended-field RT. (11.1% vs. 23.8%) [12]. This finding suggested that the aggressiveness of treatment is associated with a higher risk of HZ [12, 13]. Ramirez-Fort et al. [14] proposed that RT could augment the risk of HZ. Therefore, antiviral therapy could be considered for those with human herpes virus infection. In addition, serologic testing can help discriminate herpetic infection from RT-related necrosis. Nevertheless, some studies have had different results [8, 10]. A retrospective report revealed that RT could mitigate the risk of HZ in solid cancers (aHR: 0.94, *p* < 0.001) [8]. Our data indicated that RT advanced HZ development. Nevertheless, further studies are warranted to clarify this.

Regarding other treatment-related factors, CT has been considered a contributing factor to a high risk of HZ [5, 6, 15–17]. However, this finding must be further validated since the risk with cancer itself or with treatment modalities did not be adjusted in most previous studies. A retrospective analysis revealed that the incidence rate in 1768 solid cancer patients who received CT was 5.1% [15]. So far, it is not clear whether CT and RT have a synergistic effect on the occurrence of HZ. Our study revealed for the first time that patients with HN cancer being treated with CT and RT had the highest risk of HZ (20.84 per 1000 PYs), which was more than two times higher than normal controls (aHR: 2.46, *p* < 0.0001). For those who received RT alone, there was only a trend of higher incidence (aHR: 1.61, *p* = 0.09) No difference existed between the general population and those with CT alone or without RT or CT (S5 Table). Nevertheless, Qian et al. [6] conducted a similar analysis and came to a different conclusion. The aHRs of HZ were 1.1 for those who received neither CT nor RT, 1.38 for the group of RT alone, 1.81 for the group of CT and RT, and 1.84 for the group of CT alone. Individuals with neither CT nor RT had the same risk of HZ as the general population. Among those receiving CT, RT was not associated with HZ development. Therefore, they concluded that the increased risk might be attributed to CT [6]. However, we found CT itself had no effect on HZ (aHR: 1.47, *p* = 0.15), and the synergistic effect of CT was observed only in HN cancer patients who received RT. Contrastingly, in our previous work on gynecological cancer, we established that both CT and RT had significant roles in HZ development [18].

Certain causative factors in the development of HZ have been proposed, including advanced age, HIV [19], DM [20, 21], organ transplantation [22, 23], and cancer treatments [24]. The relationship between cancer itself or specific treatment modalities and the risk of HZ is not fully understood [5–7, 21, 24, 25]. The age- and sex-standardized rates of HZ were evaluated by Habel et al. [5] Compared with individuals without cancer, the HR of HZ was 4.8 and 1.9 for those with hematologic malignancies and solid tumors, respectively. The same conclusion was drawn by another study, which showed that the aHRs of HZ was 3.74 for hematological malignancy and 1.3 for solid cancers [6]. Apart from this, the risk of HZ was assessed in patients with 21 common malignancies in a population-based cohort. Overall, malignancy was a significant risk factor of HZ (aHR: 1.29), especially hematological cancer (aHR 2.46). Among patients with HN cancer, only those with oral cancer presented with a significantly high risk of HZ (aHR: 1.41, *p* = 0.006). There was no difference in patients with laryngeal or salivary gland cancer [7]. A strong association was also found between cancer and HZ in childhood cancer survivors. Lin et al. [26] concluded that children with cancer had a higher risk of HZ than those without cancer (cumulative incidence: 20.7 vs. 2.4 per 10,000 PY; CI = 4.8–15.6, *p* < 0.0001).

Among the potential confounding factors, RA had an impact on the onset of HZ (aHR: 2.87, *p* = 0.02). Similarly, a retrospective chart review showed that patients with RA were more likely to develop HZ than those without RA (12.1 vs. 5.4 per 1000 PYs, aHR: 2.4). In the RA group, those with erosive disease, previous joint surgery, and hydroxychloroquine or corticosteroids use had a significantly higher risk of HZ [27]. Of note, a lower prevalence of RA was consistently noted in Asia than in the United States and Europe, with a rate of approximately 0.2%–0.3% in China and 0.8% in western countries [28]. Although RA is a rare disease, patients with the above-mentioned factors are particularly at risk of HZ. Health care professionals should pay more attention to these groups of patients.

The current study emphasized the time trends of HZ development and HZ with complications in the RT and non-RT cohorts. Because HZ is commonly observed in the radiation field, the rate of HZ-related complications in the central nervous system and the HN region merits further survey. Morbidities in these regions, including encephalitis, blindness, deafness, and refractory neuralgia, can be severe [29]. Surprisingly, the occurrence of these morbidities was quite low in our cohort (3 in the RT vs. 0 in the non-RT cohort). The time trends should serve as a warning for physicians regarding the onset of HZ in RT recipients during the follow-up period, particularly within 2 years.

Some weakness in our study should be stated. First, other potential confounding factors like cancer stage, family history, or body mass index were not taken into account. Second, no data were available on the treatment intent of RT, irradiated site, dose, and volume. Third, the anatomical location of HZ could not be ascertained. Data on these factors were unavailable in the NHIRD. Future studies with detailed treatment-related information and personal risk factors must be performed.

## Conclusions

In summary, HZ is a highly morbid condition that can be triggered by RT in patients with HN cancer, with 2-year and 5-year incidence rate of 4.8% and 8.9%. The risk of HZ increased and persisted within the first 2 years and decreased thereafter. Accordingly, surveillance of HZ development among patients with HN cancer receiving RT is important to improve the quality of health care.

## Supporting information

S1 Fig Flow chart for case and control selection. LHID, Longitudinal Health Insurance Database.
(DOCX)

S2 Fig Survival analysis of the radiotherapy and non-radiotherapy cohort. RT, radiotherapy.
(DOCX)

S1 Table Details of ICD-9-CM code for head and neck cancers. (DOCX)

S2 Table Two groups for ICD-9-CM codes of herpes zoster according to complications. (DOCX)

S3 Table The risk of herpes zoster among general population and HN cancer patients. (DOCX)

S4 Table Analysis of herpes zoster with and without complications in CNS and HN regions. (DOCX)

S5 Table The risk of herpes zoster associated with the combination of RT and CT compared with the general population. (DOCX)
