# Supplementary figures and images for: Incidence and time trends of herpes zoster among patients with head and neck cancer who did and did not undergo radiotherapy: A population-based cohort study
Source: PLoS One. 2021 May 20;16(5):e0250724. doi: 10.1371/journal.pone.0250724 (PMC8136642; doi:10.1371/journal.pone.0250724)

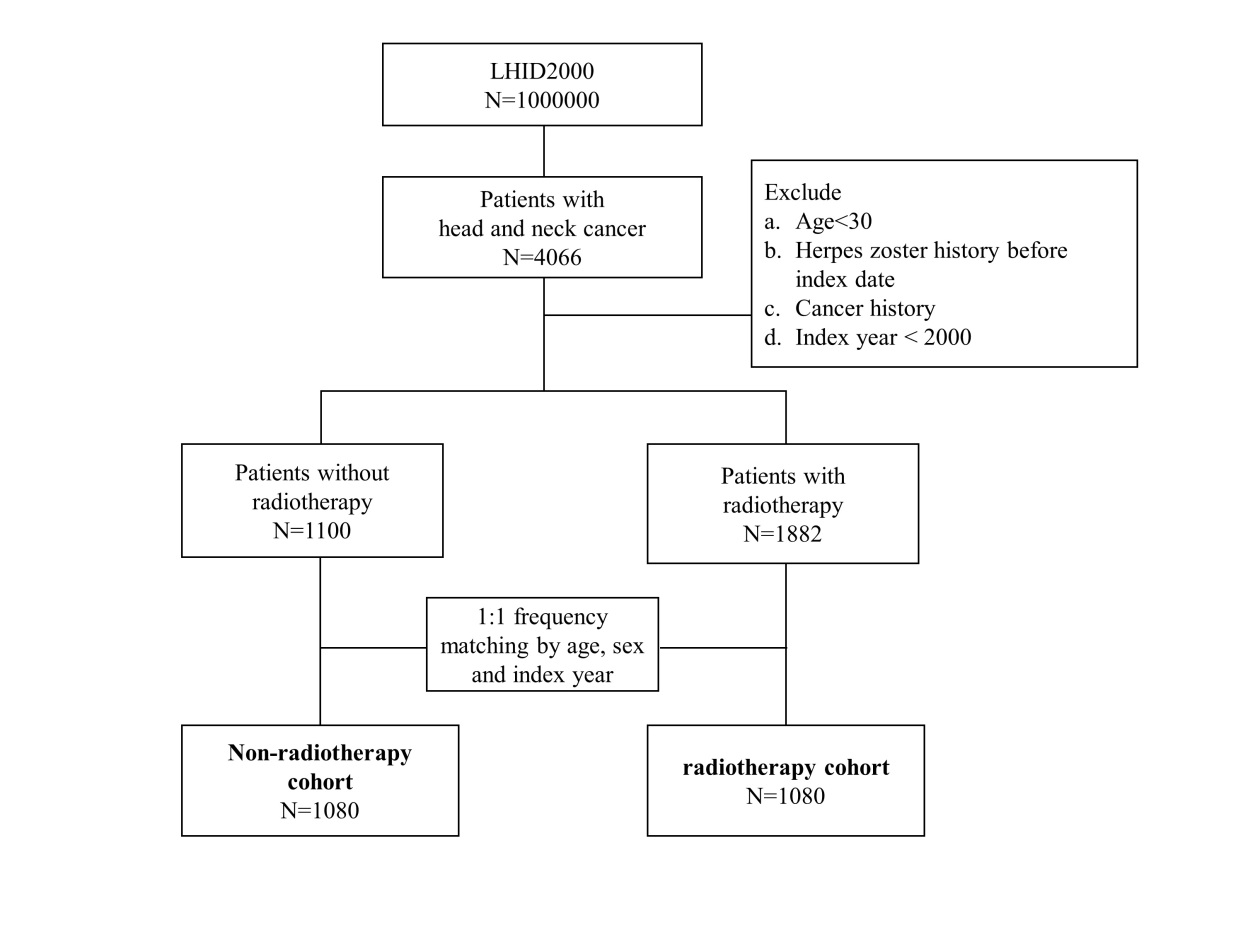

Supplement: S1 Fig — LHID, Longitudinal Health Insurance Database. (DOCX) [file pone.0250724.s001.docx]

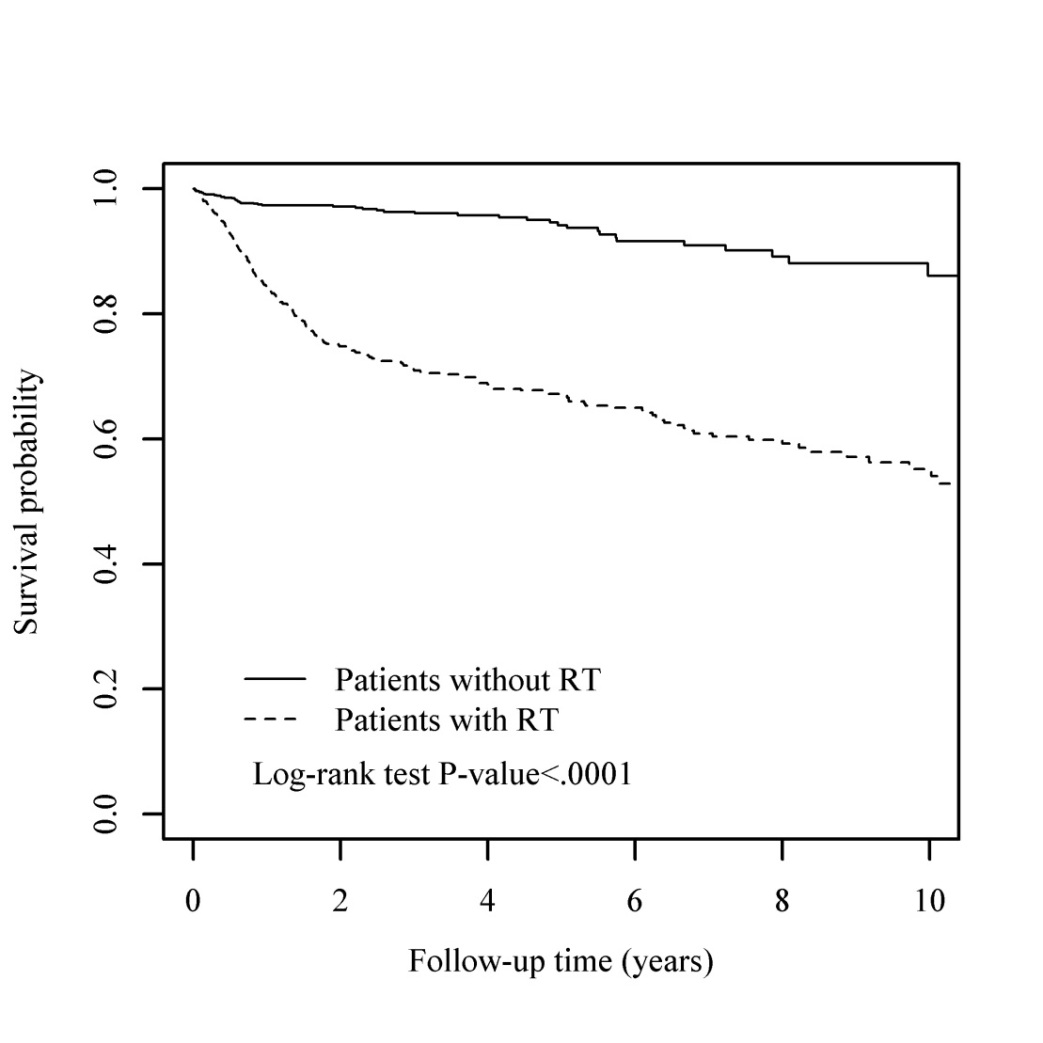

Supplement: S2 Fig — RT, radiotherapy. (DOCX) [file pone.0250724.s002.docx]
