# Supplementary material for: Incidence and time trends of herpes zoster among patients with head and neck cancer who did and did not undergo radiotherapy: A population-based cohort study
Source: PLoS One. 2021 May 20;16(5):e0250724. doi: 10.1371/journal.pone.0250724 (PMC8136642; doi:10.1371/journal.pone.0250724)
Supplement: S1 Table — (DOCX) [file pone.0250724.s003.docx]

**S1 Table. Details of ICD-9-CM code for head and neck cancers**

| [140](http://www.icd9data.com/2012/Volume1/140-239/140-149/140/default.htm) | malignant neoplasm of lip |
| --- | --- |
| [141](http://www.icd9data.com/2012/Volume1/140-239/140-149/141/default.htm) | malignant neoplasm of tongue |
| [142](http://www.icd9data.com/2012/Volume1/140-239/140-149/142/default.htm) | malignant neoplasm of major salivary glands |
| [143](http://www.icd9data.com/2012/Volume1/140-239/140-149/143/default.htm) | malignant neoplasm of gum |
| [144](http://www.icd9data.com/2012/Volume1/140-239/140-149/144/default.htm) | malignant neoplasm of floor of mouth |
| [145](http://www.icd9data.com/2012/Volume1/140-239/140-149/145/default.htm) | malignant neoplasm of other and unspecified parts of mouth |
| [146](http://www.icd9data.com/2012/Volume1/140-239/140-149/146/default.htm) | malignant neoplasm of oropharynx |
| [147](http://www.icd9data.com/2012/Volume1/140-239/140-149/147/default.htm) | malignant neoplasm of nasopharynx |
| [148](http://www.icd9data.com/2012/Volume1/140-239/140-149/148/default.htm) | malignant neoplasm of hypopharynx |
| [149](http://www.icd9data.com/2012/Volume1/140-239/140-149/149/default.htm) | malignant neoplasm of other and ill-defined sites within the lip oral cavity and pharynx |
| [160](http://www.icd9data.com/2012/Volume1/140-239/160-165/160/default.htm) | malignant neoplasm of nasal cavities middle ear and accessory sinuses |
| [161](http://www.icd9data.com/2012/Volume1/140-239/160-165/161/default.htm) | malignant neoplasm of larynx |
| [195.0](http://www.icd9data.com/2012/Volume1/140-239/190-199/195/195.0.htm) | malignant neoplasm of head, face, and neck |
| [196.0](http://www.icd9data.com/2012/Volume1/140-239/190-199/196/196.0.htm) | secondary and unspecified malignant neoplasm of lymph nodes of head, face, and neck |
