# Supplementary material for: Incidence and time trends of herpes zoster among patients with head and neck cancer who did and did not undergo radiotherapy: A population-based cohort study
Source: PLoS One. 2021 May 20;16(5):e0250724. doi: 10.1371/journal.pone.0250724 (PMC8136642; doi:10.1371/journal.pone.0250724)
Supplement: S2 Table — (DOCX) [file pone.0250724.s004.docx]

**S2 Table. Two groups for ICD-9-CM codes of herpes zoster according to complications**

| Herpes zoster with complications in central nervous system or head and neck region | |
| --- | --- |
| 053.0 | herpes zoster with meningitis |
| 053.11 | geniculate herpes zoster |
| 053.12 | postherpetic trigeminal neuralgia |
| 053.20 | herpes zoster dermatitis of eyelid |
| 053.21 | herpes zoster keratoconjunctivitis |
| 053.22 | herpes zoster iridocyclitis |
| 053.29 | herpes zoster with other ophthalmic complication |
| 053.71 | Otitis externa due to herpes zoster |
| Herpes zoster with other complications or without complications | |
| 053.10 | herpes zoster with unspecified nervous system complication |
| 053.13 | postherpetic polyneuropathy |
| 053.19 | herpes zoster with other nervous system complications |
| 053.79 | herpes zoster with other specified complications |
| 053.8 | herpes zoster with unspecified complication |
| 053.9 | herpes zoster without mention of complication |
