# Supplementary material for: Incidence and time trends of herpes zoster among patients with head and neck cancer who did and did not undergo radiotherapy: A population-based cohort study
Source: PLoS One. 2021 May 20;16(5):e0250724. doi: 10.1371/journal.pone.0250724 (PMC8136642; doi:10.1371/journal.pone.0250724)
Supplement: S3 Table — (DOCX) [file pone.0250724.s005.docx]

| **S3 Table. The risk of herpes zoster among general population and HN cancer patients** | | | | |  |  | |  | |
| --- | --- | --- | --- | --- | --- | --- | --- | --- | --- |
| **Patients group** | **Herpes zoster (n = 128)** | | | **Crude HR  (95% CI)** | | **p-value** | **Adjusted HR (95% CI)** | | **p-value** |
|  | **Event** | **PY** | **IR** |  |  |  |  |  |  |
| General population | 43 | 5334 | 8.06 | 1 (reference) | |  | 1 (reference) | |  |
| All HN cancer | 85 | 6220 | 13.67 | 1.63 (1.13–2.36)* | | 0.009 | 1.27 (0.82–2.00) | | 0.18 |
| HN cancer without RT | 29 | 3201 | 9.06 | 1.08 (0.67–1.72) | | 0.76 | 0.89 (0.53–1.48) | | 0.66 |
| HN cancer with RT | 56 | 3019 | 18.55 | 2.23 (1.49–3.32)* | | <0.0001 | 1.51 (0.92–2.79) | | 0.17 |

*p < 0.05

PY, person-years; IR, incidence rate, per 1000 PY; HR, hazard ratio; CI, confidence interval; HN, head and neck

aHR adjusted for age, sex, hypertension, diabetes mellitus, hepatitis B, hepatitis C, systemic lupus erythematosus, rheumatoid arthritis, COPD, oncological surgery and chemotherapy drugs
