# Supplementary material for: Incidence and time trends of herpes zoster among patients with head and neck cancer who did and did not undergo radiotherapy: A population-based cohort study
Source: PLoS One. 2021 May 20;16(5):e0250724. doi: 10.1371/journal.pone.0250724 (PMC8136642; doi:10.1371/journal.pone.0250724)
Supplement: S4 Table — (DOCX) [file pone.0250724.s006.docx]

**S4 Table. Analysis of herpes zoster with and without complications in CNS and HN regions**

| **Variables** | **Herpes zoster with complications  in CNS and HN region (n = 3)** | | | **Crude HR  (95% CI)** | **p-value** | **Adjusted HR (95% CI)** | **p-value** |
| --- | --- | --- | --- | --- | --- | --- | --- |
|  | **Event** | **PY** | **IR** |  |  |  |  |
| **Radiotherapy** |  |  |  |  | -- |  | -- |
| No | 0 | 3201 | 0.00 | 1 (reference) |  | 1 (reference) |  |
| Yes | 3 | 3019 | 0.99 | -- |  | -- |  |
| **Variables** | **Herpes zoster with other complications or no complications (n = 82)** | | | **Crude HR  (95% CI)** | **p-value** | **Adjusted HR (95% CI)** | **p-value** |
|  | **Event** | **PY** | **IR** |  |  |  |  |
| **Radiotherapy** |  |  |  |  |  |  |  |
| No | 29 | 3201 | 9.06 | 1 (reference) |  | 1 (reference) |  |
| Yes | 53 | 3019 | 17.56 | 2.00 (1.27–3.14)* | 0.003 | 1.71 (1.02–2.95)* | 0.04 |

*p < 0.05

PY, person-years; IR, incidence rate, per 1000 PY; HR, hazard ratio; CI, confidence interval; CNS, central nervous system; HN, head and neck

aHR adjusted for age, sex, hypertension, diabetes mellitus, hepatitis B, hepatitis C, systemic lupus erythematosus, rheumatoid arthritis, COPD, oncological surgery and chemotherapy drugs
