# Supplementary material for: Incidence and time trends of herpes zoster among patients with head and neck cancer who did and did not undergo radiotherapy: A population-based cohort study
Source: PLoS One. 2021 May 20;16(5):e0250724. doi: 10.1371/journal.pone.0250724 (PMC8136642; doi:10.1371/journal.pone.0250724)
Supplement: S5 Table — (DOCX) [file pone.0250724.s007.docx]

| **S5 Table. The risk of herpes zoster associated with the combination of RT and CT compared with the general population** | | | | | | | |
| --- | --- | --- | --- | --- | --- | --- | --- |
| **Patients group** | **Herpes zoster (n = 128)** | | | **Crude HR  (95% CI)** | **p-value** | **Adjusted HR (95% CI)** | **p-value** |
|  | **Event** | **PY** | **IR** |  |  |  |  |
| General population | 43 | 5334 | 8.06 | 1 (reference) |  | 1 (reference) |  |
| Patients without RT and CT | 27 | 2959 | 9.12 | 1.08 (0.67–1.76) | 0.74 | 1.00 (0.61–1.61) | 0.89 |
| Patients with RT alone | 15 | 1052 | 14.26 | 1.76 (1.02–3.17)* | 0.03 | 1.61 (0.98–2.89) | 0.09 |
| Patients with CT alone | 2 | 241 | 8.30 | 0.98 (0.26–4.06) | 0.88 | 0.94 (0.22–3.92) | 0.94 |
| Patients with RT and CT | 41 | 1967 | 20.84 | 2.48 (1.61–3.81)* | <0.0001 | 2.46 (1.59–3.79)* | <0.0001 |

*p < 0.05

PY, person-years; IR, incidence rate, per 1000 PY; HR, hazard ratio; CI, confidence interval; RT, radiotherapy; CT, chemotherapy

aHR adjusted for age, sex, hypertension, diabetes mellitus, hepatitis B, hepatitis C, systemic lupus erythematosus, rheumatoid arthritis, COPD, oncological surgery and chemotherapy drugs
